# Entrectinib resistance mechanisms in *ROS1*-rearranged non-small cell lung cancer

**DOI:** 10.1007/s10637-019-00795-3

**Published:** 2019-05-24

**Authors:** Bo Mi Ku, Yeon Hee Bae, Kyoung Young Lee, Jong-Mu Sun, Se-Hoon Lee, Jin Seok Ahn, Keunchil Park, Myung-Ju Ahn

**Affiliations:** 1 Research Institute for Future Medicine, Samsung Medical Center, Sungkyunkwan University School of Medicine, Seoul, South Korea; 2 Division of Hematology and Oncology, Department of Medicine, Samsung Medical Center, Sungkyunkwan University School of Medicine, 81 Irwon-ro, Gangnam-gu, Seoul, 06351 South Korea

**Keywords:** Entrectinib, ROS1, Acquired resistance, KRAS, MEK inhibitor

## Abstract

Entrectinib is a pan-tyrosine-kinase inhibitor that targets oncogenic rearrangements in *NTRK*, *ROS1* and *ALK*. The combined results of two clinical trials demonstrated the efficacy of entrectinib in *ROS1*-rearranged NSCLC. Because the development of drug resistance is inevitable, it would be helpful to determine the mechanisms of entrectinib resistance in a *ROS1*-rearranged tumor model so that future therapeutic strategies can be developed. Here, we characterized the molecular basis of resistance in entrectinib-resistant *ROS1*-rearranged HCC78 cells (HCC78ER cells). These cells were analyzed by next-generation sequencing and genetic profiling, which revealed the acquisition of *KRAS* G12C and the amplification of *KRAS* and *FGF3*. However, there were no secondary mutations in the ROS1 kinase domain. We also found that sustained ERK activation was involved in entrectinib resistance, and that combined treatment with selumetinib resensitized HCC78ER cells to entrectinib in cell viability and colony formation assays. Our data suggest that activation of the RAS signaling pathway can cause entrectinib resistance in *ROS1*-rearranged NSCLC, and is unlikely to be overcome by sequential single agent ROS1-targeting strategies against such tumors. Instead, co-targeting ROS1 and MEK may be an effective strategy for overcoming entrectinib resistance in *ROS1*-rearranged NSCLC.

**Electronic supplementary material:**

The online version of this article (10.1007/s10637-019-00795-3) contains supplementary material, which is available to authorized users.

## Introduction

The treatment of non-small cell lung cancer (NSCLC) has been revolutionized by the therapeutic targeting of oncogenic mutations, and tyrosine kinase inhibitors (TKIs) have emerged as a particularly successful treatment modality [1–3]. However, acquired resistance to TKIs is inevitable, and various resistance mechanisms have been discovered. Thus, the present challenge in TKI-based targeted therapy is to identify and understand the mechanisms causing resistance and to develop treatment strategies that circumvent these obstacles.

ROS1 is a receptor tyrosine kinase (RTK) that is not usually expressed at high levels in normal lung tissue. The wild-type function of ROS1 is unknown, and a natural ligand has not been identified. *ROS1* gene rearrangements occur in ~1–2% of patients with NSCLC, and have also been identified in colorectal, gastric and ovarian cancers, glioblastoma and cholangiocarcinoma [4–7]. Various partners for *ROS1* have been identified, including *CD74*, *SLC34A1*, *EZR*, *SDC4*, *FIG*, *TPM3*, *LRIG3* and *CCDC6* [5, 8, 9]. *ROS1* rearrangements with oncogenic potential have been found to constitutively activate ROS1 signaling, and although it remains unclear exactly how the ROS1 fusion proteins are activated, the PI3K/AKT, MAPK/ERK and JAK/STAT3 pathways are known to be involved. Thus, *ROS1*-rearranged NSCLCs are ‘addicted’ to ROS1 for cell growth and survival. Knocking down or pharmacologically inhibiting ROS1 has been reported to inhibit growth or induce apoptosis in *ROS1*-rearranged cell lines [5].

Crizotinib has been approved for patients with advanced *ROS1*-rearranged NSCLC, based on its dramatic improvement of the overall response rate (ORR) and progression-free survival (PFS) rate [10]. Although crizotinib is currently the only approved drug for the treatment of *ROS1*-rearranged NSCLC, several additional drugs are in clinical trials, including entrectinib, ceritinib and lorlatinib. Entrectinib is a potent oral inhibitor of the tyrosine kinases *NTRK*1/2/3, *ROS1* and *ALK* [11]. In two phase-I studies of patients with advanced or metastatic solid tumors harboring *NTRK1/2/3*, *ROS1* and *ALK* gene rearrangements, entrectinib demonstrated robust antitumor activity with fast and durable responses in TKI-naïve patients, along with substantial intracranial activity [12]. Of note, the ORR was 86% in 14 *ROS1*-rearranged solid tumors, and 13 patients who responded had *ROS1*-rearranged NSCLC [12]. Recent integrated analysis of three phase-I/II studies (STARTRK-2, STARTRK-1, and ALKA-372-001) showed deep and durable systemic response in patients with *ROS1*-rearranged NSCLC treated with entrectinib regardless of CNS metastases [13].

Although entrectinib resulted in durable disease control and prolonged PFS in *ROS1*-rearranged NSCLC [11–13], experience with targeted therapy suggests that resistance to entrectinib may also emerge and potentially limit its effectiveness. In fact, despite the promising efficacy of entrectinib, acquired resistance to entrectinib has already been reported in *NTRK*-rearranged tumors. Acquired entrectinib resistance was mediated by *NTRK1* G595R and G667C mutations in a patient with metastatic colorectal carcinoma harboring an *LMNA-NTRK1* rearrangement, and by an *NTRK3* G623R mutation in a patient with mammary analogue secretory carcinoma (MASC) harboring an *ETV6-NTRK3* rearrangement [14, 15]. However, the mechanism of acquired resistance to entrectinib remains to be determined in *ROS1*-rearranged NSCLC.

Our aim in this study was to discover the acquired resistance mechanisms in *ROS1*-rearranged NSCLC using an in vitro entrectinib resistance induction model. We identified the *KRAS* G12C mutation and sustained ERK activation as mechanisms of entrectinib resistance.

## Materials and methods

### Cell lines, antibodies and reagents

HCC78 cell was obtained from the Deutsche Sammlung von Mikroorganismen und Zellkulturen (DSMZ) and cultured in RPMI-1640 medium supplemented with 10% FBS, penicillin (100 U/mL) and streptomycin (100 μg/mL) at 37 °C in a humidified atmosphere containing 5% CO_2_. Cell line identity was authenticated by short-tandem-repeat analysis. Entrectinib-resistant HCC78 (HCC78ER) cells were newly established in our laboratory through the exposure of HCC78 cells to gradually increasing concentrations of entrectinib (starting at 100 nM and ending with 5 μM) over 6 months. The established cells maintained resistance to entrectinib even after the withdrawal of entrectinib from the culture medium.

Entrectinib was provided by Ignyta, Inc./F.Hoffmann-La Roche Ltd. Crizotinib, ceritinib, lorlatinib and selumetinib were purchased from Selleckchem. All drugs were dissolved at a 10 mM concentration in dimethyl sulfoxide (DMSO) and stored in small aliquots at −20 °C until further use. Antibodies specific for p-ROS1 (Tyr1068), ROS1, p-AKT (Ser473), AKT, p-ERK1/2 (Thr202/Tyr204), ERK1/2, p-STAT3 (Tyr705), STAT3, p-p53 (Ser15), p53, p-H2AX (Ser139), H2AX and PARP were obtained from Cell Signaling Technologies. Anti-FGF3 and β-actin antibodies were obtained from Santa Cruz Biotechnology.

### Cell viability assay

Cells were seeded on a 96-well plate, allowed to adhere overnight, and treated with the indicated drugs for 72 h. Cell viability was determined with a Cell Counting Kit-8 (Dojindo Molecular Technologies) according to the manufacturer’s instructions. Long-term viability was assessed with a colony formation assay. In brief, cells were seeded in 24-well plates. Following 10–14 days of treatment, the cells were fixed and stained with crystal violet.

### Cell proliferation assay

Cells were seeded on a 96-well plate, allowed to adhere overnight, and treated with the indicated drugs for 24 h. Cell proliferation was determined with a BrdU cell proliferation assay kit (Cell Signaling Technologies) according to the manufacturer’s instructions.

### Western blotting

Cells were lysed in NP-40 lysis buffer supplemented with a protease and phosphatase inhibitor cocktail (Sigma). Equal amounts of protein were subjected to SDS-PAGE and transferred to polyvinylidene difluoride (PVDF) membranes. After being blocked in 5% skim milk, the membranes were sequentially incubated with the indicated primary antibodies and the appropriate secondary antibodies, and were developed by ECL. For proteome profiler array, the Human XL Oncology Array Kit (R&D Systems) was used for the parallel determination of relative levels of 84 human cancer-related proteins.

### Genetic analysis

Next-generation sequencing (NGS) analysis was performed on a targeted sequencing platform (CancerSCAN™) designed at Samsung Medical Center [16]. The CancerSCAN™ panel is designed to target 375 cancer-related genes. Genomic DNA (250 ng) was sheared in a Covaris S220 ultrasonicator (Covaris, Woburn, MA, USA), and target-capture was performed with the SureSelect XT reagent kit, HSQ (Agilent Technologies) according to the manufacturer’s protocol.

After enriched exome libraries were multiplexed, the libraries were sequenced on a HiSeq 2500 sequencing platform (Illumina). Briefly, a paired-end DNA sequencing library was prepared through gDNA shearing, end-repair, A-tailing, paired-end adaptor ligation and amplification. After hybridization of the library with bait sequences for 27 h, the captured library was purified and amplified with an index barcode tag, and the library quality and quantity were assessed. The exome library was sequenced via the 100-bp paired-end mode of the TruSeq Rapid PE Cluster Kit and the TruSeq Rapid SBS Kit (Illumina).

Sequence reads were mapped to the human genome (hg19) by means of Burrows-Wheeler Aligner (BWA). Duplicate read removal was performed with Picard and SAMtools. Local alignment was optimized with the Genome Analysis Toolkit (GATK). Variant calling (SNVs, small indels, CNVs and gene fusion) was done only in regions targeted in CancerSCAN*™*.

### Statistical analysis

Data are presented as the mean ± SE. Statistical analyses were carried out in GraphPad Prism (GraphPad software). Statistical evaluation was performed with a two-tailed Student’s t test, and *P* values <0.05 were considered statistically significant.

## Results

### Entrectinib treatment inhibited cell survival and induced apoptosis in *ROS1*-rearranged cells

**Fig. 1:**
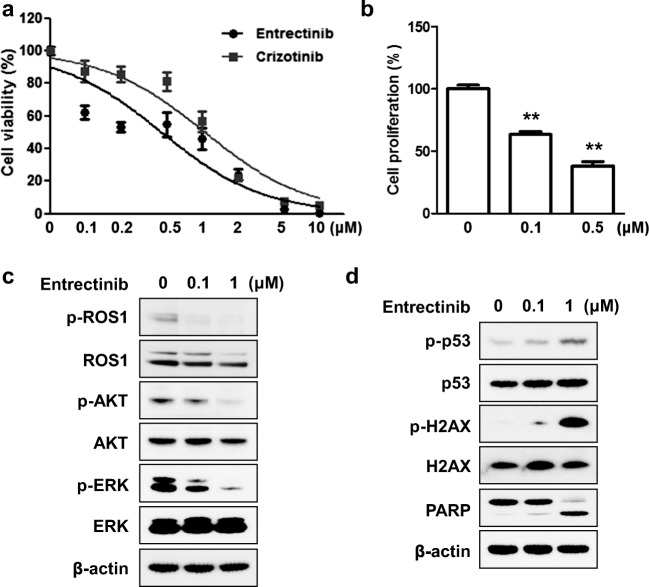
Entrectinib treatment inhibits cell survival and induces apoptosis in HCC78 cells harboring SLC34A2-ROS1. **a** HCC78 cells were treated with the indicated concentrations of entrectinib or crizotinib for 72 h. Cell viability was determined using CCK-8 assay and data are presented as mean ± SE (*n* = 6). **b** After 24 h entrectinib treatment, cell proliferation was measured using BrdU incorporation assay. Data are presented as mean ± SE (*n* = 9). **, *P* < 0.01. **c** HCC78 cells were treated with indicated concentrations of entrectinib for 24 h. Inhibition of ROS-1 and its downstream signaling AKT and ERK were confirmed by Western blotting. **d** After 24 h entrectinib treatment, cell lysates were analyzed by Western blotting to assess DNA damage and PARP cleavage. β-actin was used as a loading control

*SLC34A2-ROS1* fusion was identified as potential driver mutation in HCC78 cells. HCC78 cells were used as in vitro model system for *ROS1*-rearrnaged NSCLC [6, 17, 18]. Previously, activation of EGFR or c-MET signaling has been reported in HCC78 cells suggesting inhibition of ROS1 pathway only might not be effective in vitro model system [19–21]. Therefore, to evaluate whether the parental HCC78 cells are also dependent upon other receptor tyrosine kinase such as EGFR or c-MET, we conducted proteome profiler array using the Human XL Oncology Array Kit. In our experiment conditions, EGFR and c-MET expressions were barely detected in HCC78 cells (Supplementary Fig. 1). Thus, we used HCC78 cells as *ROS1*-driven NSCLC model system to investigate the effects of entrectinib on *ROS1*-rearranged NSCLC. Entrectinib inhibited cell survival more effectively than crizotinib, a drug that is known to inhibit the growth of HCC78 cells [6, 17, 18] (Fig. 1a). The IC_50_ value (450 nM) of entrectinib for inhibition of HCC78 is similar to that reported previously [18]. Cell proliferation was also significantly inhibited by entrectinib treatment (Fig. 1b). To explore the biologic activity of entrectinib in HCC78 cells dependent on ROS1 kinase, we conducted Western blotting. Entrectinib inhibited the phosphorylation of ROS1 and its downstream signaling proteins AKT and ERK (Fig. 1c). In addition, entrectinib treatment induced DNA damage and apoptotic cell death, as evidenced by the expression of p-p53, p-H2AX and cleaved PARP (Fig. 1d). These results suggest that entrectinib induces cytotoxicity by inhibiting the ROS1 signaling pathway and inducing apoptosis.

### Entrectinib-resistant HCC78 cells had the *KRAS* G12C mutation

**Fig. 2:**
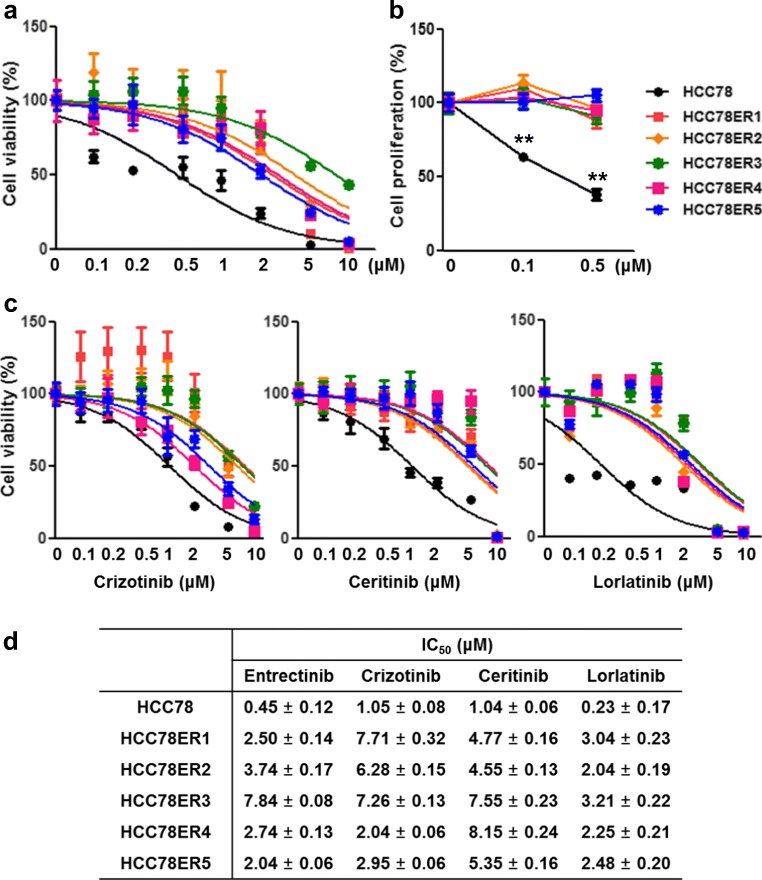
Entrectinib-resistant HCC78 cells exhibit cross-resistance to other ROS1 inhibitors. **a** HCC78 and HCC78ER cells were treated with the indicated concentrations of entrectinib for 72 h. Cell viability was determined using CCK-8 assay and data are presented as mean ± SE (n = 6). **b** Cell proliferation was measured using BrdU incorporation at 24 h after entrectinib treatment. Data are presented as mean ± SE (*n* = 3). **, *P* < 0.01. **c** HCC78 and HCC78ER cells were treated with various concentrations of crizotinib, ceritinib, or lorlatinib for 72 h. Cell viability was measured by CCK-8 assay and presented as mean ± SE (n = 6). **d** Summary of IC_50_. The IC_50_ value was determined using nonlinear regression curve fit of GraphPad Prism

To demonstrate the entrectinib resistance mechanism in a *ROS1*-fusion-positive setting, we established entrectinib-resistant HCC78 clones (HCC78ER1–5). HCC78ER cells were resistant to entrectinib (Fig. 2a and b) and were cross-resistant to other ROS1 TKIs such as crizotinib, ceritinib and lorlatinib (Fig. 2c). The IC_50_ values were much higher in HCC78ER cells than in the parental HCC78 cells (Fig. 2d).

**Fig. 3:**
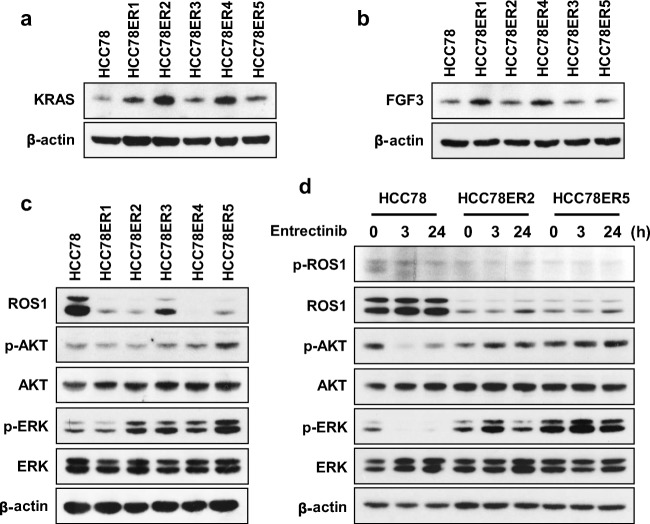
Entrectinib-resistant HCC78 cells contain KRAS G12C mutation and show sustained ERK activation after entrectinib treatment. **a-b** Relative expression levels of KRAS (a) and FGF3 (b) were compared between HCC78 and HCC78ER cells. **c** Phosphorylation of ROS1, AKT, and ERK were detected by Western blotting. **d** HCC78, HCC78ER2, and HCC78ER5 cells were treated with entrectinib (0.5 μM) for the indicated times. Relative phosphorylation of ROS-1, AKT, and ERK were assessed using western blotting. β-actin was used as a loading control

To investigate the molecular basis of acquired resistance to entrectinib, we performed next-generation sequencing (NGS) on DNA extracted from HCC78 and HCC78ER cells to detect gene mutations, fusions and copy-number variations across 375 cancer-related genes. The genetic alterations detected in our NGS analysis are summarized in Table 1. The HCC78ER clones still harbored the *SLC34A2-ROS1* fusion gene, but had no additional *ROS1* mutations. Notably, all the HCC78ER clones contained a *KRAS* G12C mutation, which was not present in the parental HCC78 cells. In addition, *KRAS* amplification was found in HCC78ER2, and *FGF3* amplification was found in HCC78ER1 and 4. These gene amplifications increased the protein expression of KRAS and FGF3 in HCC78ER cells (Fig. 3a and b).

**Table 1:** Summary of genetic alterations in HCC78 and HCC78ER cells

	Identified alterations
HCC78	*SLC34A2-ROS1*
HCC78ER1	*SLC34A2-ROS1*, *KRAS* G12C, *FGF3* amplification
HCC78ER2	*SLC34A2-ROS1*, *KRAS* G12C, *KRAS* amplification
HCC78ER3	*SLC34A2-ROS1*, *KRAS* G12C
HCC78ER4	*SLC34A2-ROS1*, *KRAS* G12C, *FGF3* amplification
HCC78ER5	*SLC34A2-ROS1*, *KRAS* G12C

Although the *SLC34A2-ROS1* fusion gene was detected with similar read counts in HCC78ER and HCC78 cells in NGS analysis, ROS1 protein expression was substantially lower in HCC78ER cells (Fig. 3c). On the other hand, the levels of phosphorylated AKT and ERK, components of oncogenic signaling pathways downstream of ROS1, were maintained or increased in HCC78ER cells compared to HCC78 cells (Fig. 3c). In agreement, entrectinib effectively inhibited the phosphorylation of ROS1, AKT and ERK in the parental HCC78 cells, but failed to do so in HCC78ER cells (Fig. 3d).

### Activation of ERK signaling was required for the survival of HCC78ER cells under entrectinib treatment

**Fig. 4:**
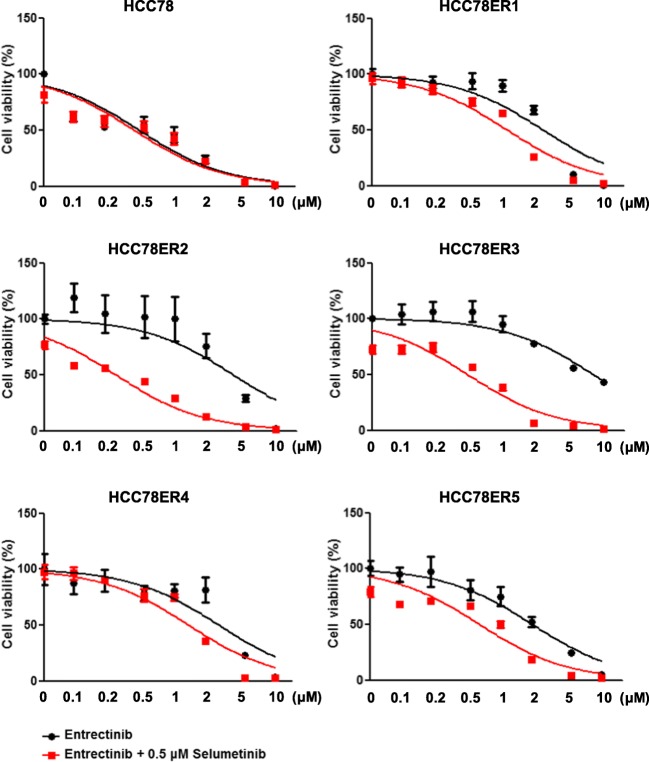
The combination of MEK inhibitor with entrectinib restores sensitivity to entrectinib in HCC78ER cells. HCC78, HCC78ER1, HCC78ER2, HCC78ER3, HCC78ER4, and HCC78ER5 cells were treated with indicated concentrations of entrectinib alone or entrectinib + selumetinib (0.5 μM) for 72 h. Cell viability was determined using CCK-8 assay. The data are mean ± SE (n = 6)

**Fig. 5:**
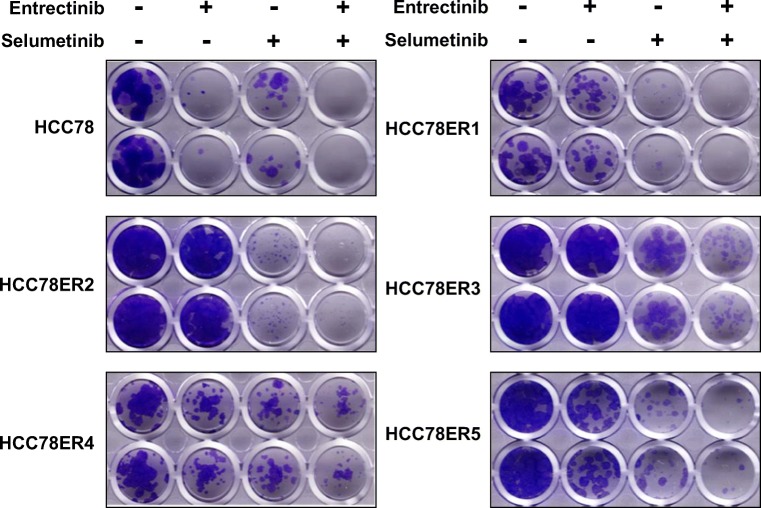
Cotargeting of ROS1 and MEK efficiently inhibits resistance to entrectinib. Cells were treated with entrectinib (0.5 μM) alone, selumetinib (0.5 μM) alone, or their combination for 10~14 days. The representative images are shown

**Fig. 6:**
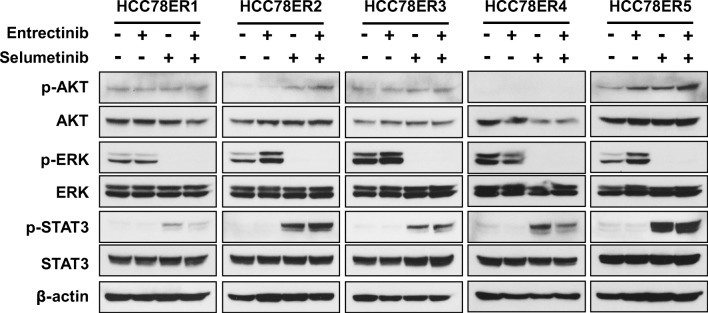
MEK inhibition activates STAT3 signaling in HCC78ER cells. Cells were treated with entrectinib (0.5 μM) alone, selumetinib (0.5 μM) alone, or their combination for 24 h. Western blotting carried out to determine the phosphorylation levels of AKT, ERK, and STAT3. β-actin was used as a loading control

To determine whether the survival of the resistant clones was the result of sustained ERK activation, we tested the anti-tumor effects of combined treatment with entrectinib and selumetinib, a MEK inhibitor. Although selumetinib alone had limited effects on the HCC78ER cells (data not shown), the combination of entrectinib with selumetinib effectively inhibited the growth of these cells, both in a short-term cell viability assay (Fig. 4) and in a long-term colony formation assay (Fig. 5). Consistent with these results, selumetinib treatment in the presence of entrectinib completely inhibited ERK phosphorylation in HCC78ER cells (Fig. 6).

Although the inhibition of ERK signaling by selumetinib increased the sensitivity of HCC78ER cells to entrectinib, it did not completely overcome entrectinib resistance. These results suggest that other resistance mechanisms existed in HCC78ER cells after selumetinib treatment. Because a previous report indicated that MEK inhibition activated STAT3 signaling in *KRAS*-mutant lung cancer cells [22], we examined the activation of STAT3 signaling. Consistent with the previous report, MEK inhibition activated STAT3 in *KRAS*-mutant HCC78ER cells (Fig. 6).

## Discussion

This is the first preclinical study reporting a mechanism of acquired entrectinib resistance in *ROS1*-rearranged NSCLC cells. In this study, entrectinib resistance was derived from the activation of a bypass signaling pathway. Resistant cells exhibited the *KRAS* G12C mutation, along with increased KRAS protein expression. In the presence of entrectinib, the resistant cells demonstrated sustained ERK activation, despite reduced expression of phospho-ROS1. Since mutations or amplifications in *KRAS* have been associated with the activation of the MAPK/ERK pathways, constitutive ERK activation in the resistant cells may have been due to the presence of an activating mutation (G12C) or the overexpression of KRAS, and thus conferred resistance to entrectinib.

The KRAS protein may confer TKI resistance not only in the case of activating mutations, but also in the event of amplification and elevated expression of the *KRAS* gene [23–26]. Similar to our results, a previous study identified the *KRAS* G12C mutation as a resistance mechanism to the ROS1 inhibitor JNJ-ROS1i-A in HCC78 cells [23]. Cargnelutti et al. suggested that activation of the RAS pathway can confer both primary and secondary resistance in ROS1-addicted cells. In addition, they demonstrated that cells with the *KRAS* G12C mutation strongly downregulated *SCL34A2-ROS1* mRNA expression [23]. Reduced expression of ROS1 has been found not only in KRAS-mediated resistance models, but also in crizotinib-resistant HCC78 cells with activated EGFR signaling [27]. Therefore, the downregulation of ROS1 seems to be a feature of resistant HCC78 cells resulting from bypass signaling activation.

As mutations within the ROS kinase domain occur in 50–60% of crizotinib-resistant *ROS1*-rearranged tumors [28], we expected to find secondary acquired *ROS1* mutations in entrectinib-resistant cells. Although 5 crizotinib resistance mutations (S1986Y/F, L2026 M, G2032R, D2033N and L2155S) in *ROS1* have been described [8], none of them was found in our entrectinib-resistant HCC78 cell model. In previous studies, entrectinib has not exhibited activity against cells with the most common crizotinib-resistant *ROS1* mutations, including L2026 M, G2032R and D2033N [29, 30]. Taken together, these results may indicate that entrectinib and crizotinib have different binding characteristics with ROS1, which may explain the lack of secondary mutations in the ROS1 kinase domain in entrectinib-resistant cells. In addition, we found that ROS1 expression was significantly decreased in HCC78ER cells. This might support the absence of resistance mutation in ROS1 kinase domain in our in vitro system.

A previous study demonstrated that the addition of a MEK inhibitor to crizotinib improved the response to treatment in vitro and in vivo in *ALK*-rearranged NSCLC models [26]. Because one of the major goals of this study was to overcome resistance to entrectinib, we evaluated the efficacy of combined treatment with entrectinib and selumetinib in entrectinib-resistant cells. After long-term treatment, the combination of entrectinib with the MEK inhibitor, selumetinib fully restored entrectinib sensitivity in HCC78ER cells. However, this combination only partially resensitized resistant cells after short-term treatment, indicating that parallel signaling pathways can be activated for cell survival.

Constitutive activation of STAT3 has been observed in a variety of tumors, including melanoma and lung, pancreatic, colorectal and ovarian cancers. Aberrant STAT3 activation contributes to cell proliferation, differentiation, migration and survival [31–33]. Several studies have demonstrated the potential role of STAT3 signaling in TKI resistance. Inhibition of MEK triggers the feedback activation of STAT3 that contributes to drug resistance in diverse oncogene-addicted cancer models. This feedback loop indicates the cross-talk between the MEK and STAT3 signaling pathways [22]. Zhao et al. also reported that STAT3 was activated following MEK inhibition in *KRAS*-mutant pancreatic and colon cancer cells [34]. Although STAT signaling has not yet emerged as a dominant driver of resistance in *ROS1*-rearranged NSCLC, our results suggest that STAT3 activation may have limited the efficacy of our combination strategy. Further studies are warranted to determine the exact function of STAT3 activation.

Many different *ROS1* fusion partner genes have been reported and *CD74-ROS1* is the most common type of *ROS1* gene rearrangement in patients with NSCLC. However, only one type of *ROS1*-rearranged (*SLC34A2-ROS1*) cell line was evaluated for resistance mechanism in this study. Therefore, the in vitro findings in this specific cellular context should be further validated from repeat biopsy of *ROS1*-rearranged NSCLC patients who progressed on entrectinib.

In conclusion, we demonstrated that molecular changes including *KRAS* mutation were associated with acquired resistance to entrectinib. The combination of entrectinib and selumetinib may be an effective strategy for treating entrectinib resistance in *ROS1*-rearranged NSCLC, when RAS activation is involved. This strategy should be further investigated in patients with *ROS1*-rearranged NSCLC whose tumors exhibit acquired resistance to entrectinib.

## Electronic supplementary material

ESM 1 (PDF 438 kb)
