# Supplementary material for: Entrectinib resistance mechanisms in ROS1-rearranged non-small cell lung cancer
Source: Invest New Drugs. 2019 May 24;38(2):360–8. doi: 10.1007/s10637-019-00795-3 (PMC7066105; doi:10.1007/s10637-019-00795-3)
Supplement: Supplementary file 1 — (PDF 438 kb) [file 10637_2019_795_MOESM1_ESM.pdf]

Supplementary Fig 1

AUTHOR ACCEPTED MANUSCRIPT

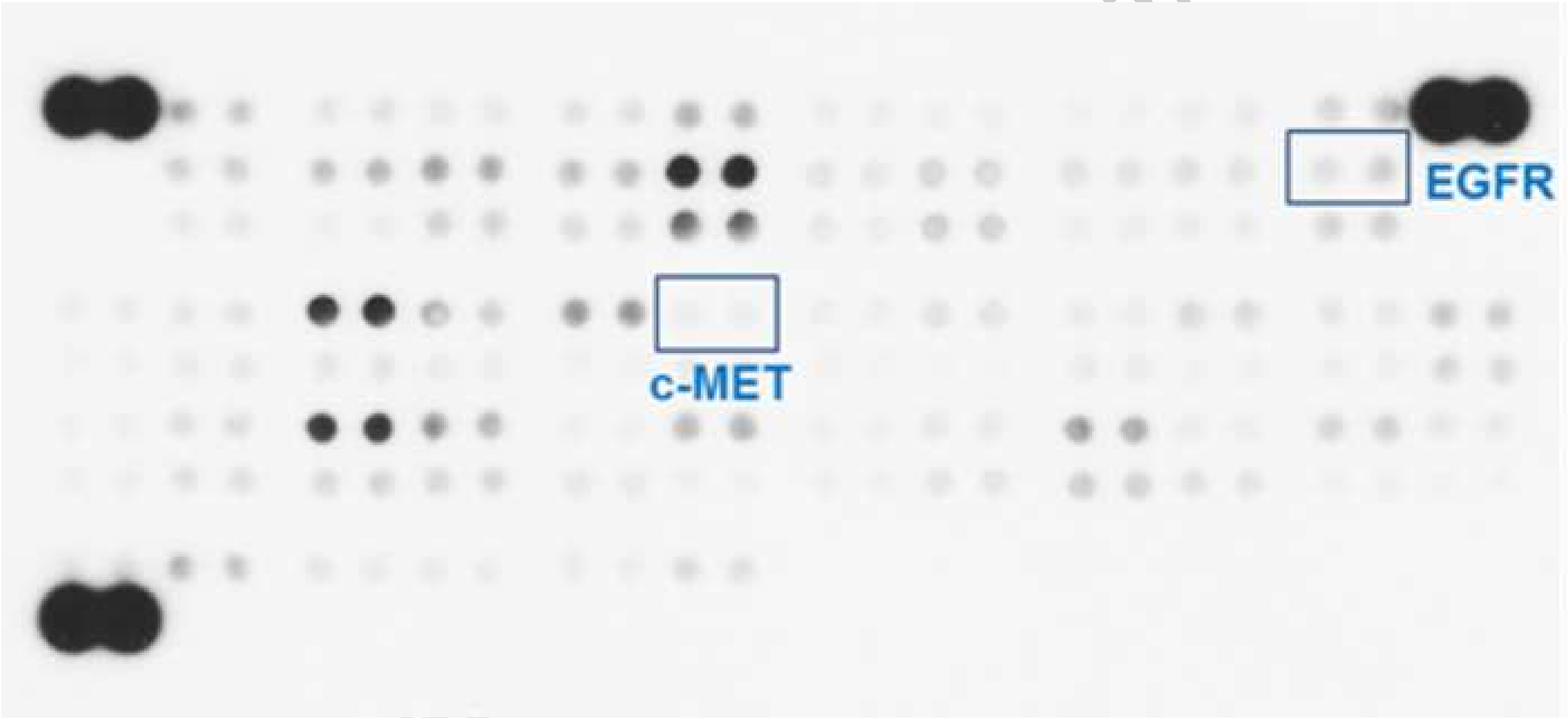

© 2019 Springer Nature B.V.

AUTHOR ACCEPTED MANUSCRIPT

Supplementary Figure legend

Supplementary Fig. 1 EGFR and c-MET expression in HCC78 cells. A human XL Oncology Array was used to determine the relative expression level of multiple oncology-related proteins in HCC78 cells.

Author accepted manuscript

© 2019 Springer Nature B.V.
